# An oxidation resistant refractory high entropy alloy protected by CrTaO_4_-based oxide

**DOI:** 10.1038/s41598-019-43819-x

**Published:** 2019-05-13

**Authors:** Kai-Chi Lo, Yao-Jen Chang, Hideyuki Murakami, Jien-Wei Yeh, An-Chou Yeh

**Affiliations:** 1 Department of Materials Science and Engineering, National Tsing Hua University, 101, Sec. 2, Kuang-Fu Road, Hsinchu, 30013 Taiwan China; 2 Research Centre for Structural Materials, National Institute for Materials Science, Sengen 1-2-1, Tsukuba, Ibaraki 305-0047 Japan; 3 Department of Nanoscience and Nanoengineering, Graduate School of Advanced Science and Engineering, Waseda University, 3-4-1 Ookubo, Shinjuku-ku, Tokyo 169-8555 Japan; 4 High Entropy Materials Center, National Tsing Hua University, 101, Sec. 2, Kuang-Fu Road, Hsinchu, 30013 Taiwan China

**Keywords:** Metals and alloys, Corrosion

## Abstract

Although refractory high entropy alloys (RHEAs) have shown potentials to be developed as structural materials for elevated temperature applications, most of the reported oxidation behaviours of RHEA were associated with short term exposures for only up to 48 hours, and there is a lack of understanding on the oxidation mechanism of any RHEA to-date. In this work, by using thermogravimetric analysis, isothermal oxidation was conducted on a novel RHEA at 1000 °C and 1100 °C for up to 200 hours, which is an unprecedented testing duration. The external oxide layer strongly influenced the weight gain behaviours, and it consisted of CrTaO_4_-based oxide with some dispersion of Al_2_O_3_ and Cr_2_O_3_. At 1000 °C, the inability to form dense CrTaO_4_-based oxide layer resulted an exponential dependence of weight gain throughout 200 hours. At 1100 °C, mass gain curve showed two parabolic dependences associated with the formation of protective CrTaO_4_-based oxide layer and the weight gain after 200 hours was 4.03 mg/cm^2^, which indicates that it is one of the most oxidation resistant RHEAs comparing to literature data to-date. This work can also provide insights on how to further develop RHEA to withstand long term oxidation at elevated temperatures.

## Introduction

As the temperature capability of modern Ni- based superalloys has reached its limit for gas turbine engine applications, materials scientists are searching for new materials with higher temperature capabilities. Recently, “High-Entropy Alloy (HEA)”^1,2^ has been proposed as a new strategy for alloy design with a wide composition space. Other names such as “compositionally complex alloys (CCAs)” has also been given to this class of alloy containing multiple phases^3^. Nevertheless, the alloy design strategy of HEA/CCA shows great promise for exploring new alloy systems. RHEA was first proposed by Senkov *et al*.^4^ as an attempt to create new alloy systems for high temperature applications beyond superalloys. By combining refractory elements such as W, Mo, Ta, Nb, Hf, …etc. in equi- or near equi-molar ratios, several RHEAs with promising mechanical properties have been reported^5–7^. However, oxidation resistance is crucial since it could lead to severe degradation in mechanical properties. For high temperature applications, formation of Al_2_O_3_^8^, Cr_2_O_3_^9^, or SiO_2_^10^ layer can provide oxidation resistance since these oxides are thermodynamically stable and exhibit low oxygen permeability. To the best of authors’ knowledge, there are only a few articles addressing the oxidation behaviours of Al, Cr, or Si-bearing RHEAs/RCCAs: Oxidation of CrMo_0.5_NbTa_0.5_TiZr alloy was firstly reported by Senkov *et al*.^11^, which showed severe spallation of oxide layer and about 120 mg/cm^2^ of mass gain after 100 hours of oxidation at 1000 °C. Gorr *et al*.^12,13^ reported the oxidation behaviour of AlCrMoTiW, AlCrMoNbTi, and (AlCrMoNbTi)_0.99_Si_0.01_ alloys. For AlCrMoTiW, despite no formation of continuous oxide layer on a sample surface was observed, the mass gain curve showed parabolic dependence, and approximately 7.9 mg/cm^2^ of mass gain was reported after 40 hours of oxidation at 1000 °C. AlCrMoNbTi possessed slightly less oxidation resistance than that of AlCrMoTiW, exhibiting oxidation mass gains of 9.2 and 8.3 mg/cm^2^ at 1000 and 1100 °C, respectively. (AlCrMoNbTi)_0.99_Si_0.01_ exhibited 5.4 and 6.4 mg/cm^2^ of mass gain at 1000 and 1100 °C after 48 hours of oxidation, respectively. The most recent study on the oxidation behaviour of AlCrMoTaTi reported by Gorr *et al*.^14^ indicated a mass gain after 48 hours of oxidation at 1000 and 1100 °C was less than 1 and 3 mg/cm^2^, respectively; this extremely low mass gain at 1000 °C was claimed to be due to formation of Al_2_O_3_ layer on the alloy surface, however, the associated oxidation mechanism was not clear since there was no detailed microstructure characterization. Furthermore, these previous oxidation studies were conducted for only up to 48 hours, and there was no indication whether this Al_2_O_3_ layer could still be effective after a longer exposure. In this work, oxidation behaviours of a novel RHEA “NV1” have been studied at 1000 and 1100 °C, with an unprecedented testing duration of 200 hours. Such oxidation study for RHEA can provide valuable information on the long-term oxidation behaviours of this class of alloy and how to improve it further.

## Results

### Pre-oxidation microstructure

**Figure 1:**
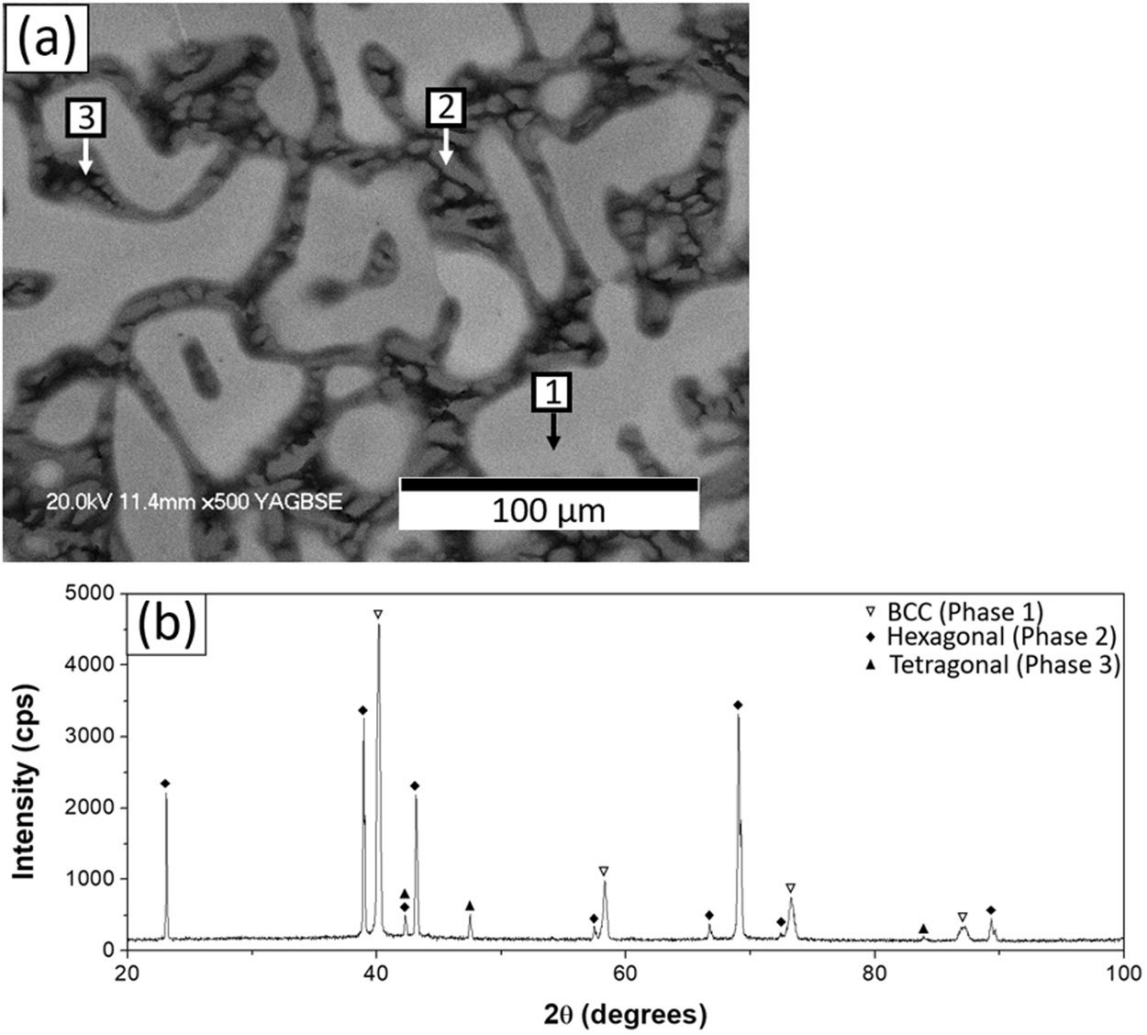
(**a**) BEI, (**b**) XRD pattern of the as-cast NV1 alloy.

The as-cast microstructure of NV1 had dendrites and contained several phases; back-scattered electron image (BEI) and the chemical composition of the observed phases are shown in Fig. 1(a) and Table 1, respectively. The dendrite core (Fig. 1(a), phase No. 1) was rich in Mo, and interdendritic regions contained Cr-rich phase (Fig. 1(a), phase No. 2) and Al-rich phase (Fig. 1(a), phase No. 3). In addition, it was found that Ti strongly segregated to Al-rich phase, while Si strongly segregated to Cr-rich phase.

**Table 1:** Chemical composition (in at%) of as-cast NV1 alloy bulk and individual phases in Fig. 1(a).

ID#	Al	Si	Ti	Cr	Nb	Mo	Ta
Bulk*	17.6 ± 0.3	2.9 ± 0.8	5.4 ± 0.3	25.2 ± 0.8	15.2 ± 0.0	20.3 ± 1.2	13.4 ± 0.9
1	15.4 ± 0.2	1.1 ± 0.0	4.6 ± 0.1	19.4 ± 0.2	14.9 ± 0.1	25.7 ± 0.3	18.9 ± 0.3
2	13.2 ± 0.2	8.4 ± 0.2	3.1 ± 0.1	40.2 ± 0.0	13.5 ± 0.1	7.8 ± 0.1	13.8 ± 0.2
3	29.6 ± 0.8	0.6 ± 0.1	15.8 ± 0.9	16.6 ± 0.5	13.2 ± 0.1	20.4 ± 1.8	3.8 ± 0.4

*Determined with X-ray fluorescence (XRF).

The XRD analysis is shown in Fig. 1(b); there are three phases present, i.e. BCC, Tetragonal, and Hexagonal phases. Image analysis was utilized to estimate fractions of each phase in Fig. 1(a). Mo-rich phase (dendrite core) had the highest volume fraction (65.44%), followed by Cr-rich phase (29.84%) and Al-rich phase (4.72%). Therefore, Phase 1 can be identified as Mo-rich BCC solid-solutioned phase with a lattice constant of 3.167 Å, which was 0.80% larger than that of pure Mo (3.142 Å)^15^. By correlating fractions of phases with the associated peak intensities, Cr-rich phase could be identified as the hexagonal Phase 2, and Al-rich phase was the tetragonal Phase 3.

### Oxidation behaviour at 1000 °C

**Figure 2:**
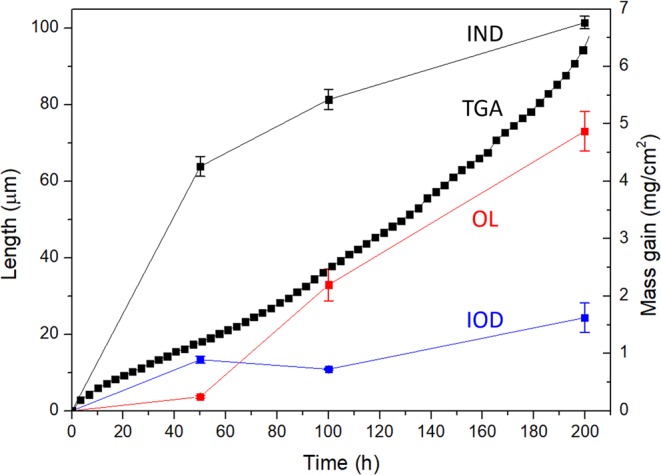
The oxidation mass gain (TGA curve) and length of OL, IOD, and IND at 1000 °C up to 200 hours. The error bars represented deviation from repeated measurements.

The oxidation mass gain curve of NV1 alloy at 1000 °C is shown in Fig. 2. After 200 hours of oxidation, the mass gain was 6.53 mg/cm^2^. The mass gain curve showed exponential dependence throughout the entire testing duration, and such dependence can be described by Eq. (1) below, where Δ*m* is the mass gain per initial surface area (mg/cm^2^) and *t* is the time elapsed (hours).

1 $${\rm{\Delta }}m=3.113{e}^{0.005t}$$

**Figure 3:**
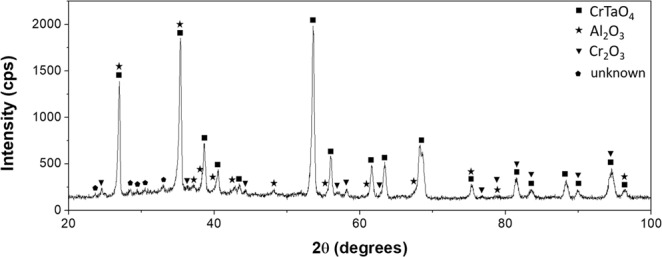
XRD pattern after 200 hours oxidation at 1000 °C.

XRD analysis on the oxidised sample surface revealed 3 different oxides existing in the oxide layer (Fig. 3): CrTaO_4_, Al_2_O_3_, and Cr_2_O_3_. Shifted peaks from CrTaO_4_ showed the highest intensity among all the observed peaks, indicating the main component of the oxide layer was CrTaO_4_-based. The detected CrTaO_4_-based oxide had lattice constants of *a* = 4.625 Å and *c* = 3.007 Å that were 0.37% less in *a* axis and 0.43% less in *c* axis comparing to those of pure CrTaO_4_ (*a* = 4.642 Å, *c* = 3.020 Å provided by PDF No. 39–1428).

**Figure 4:**
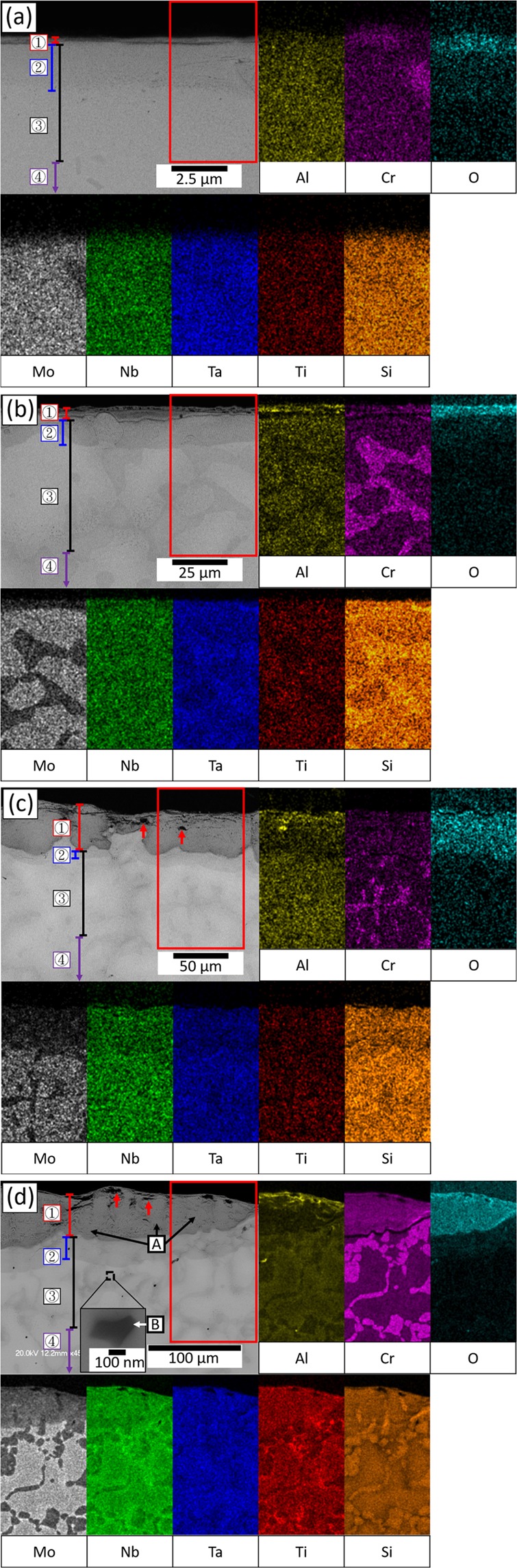
Cross-sectional BEI of the oxide layers after (**a**) 1 (**b**) 50 (**c**) 100 (**d**) 200 hours of oxidation at 1000 °C (grey scale image) and EDS mapping results of the selected region (color-coded images). 1, 2, 3, 4 respectively represent “OL”, “IOD”, “IND”, and “substrate”. The enlarged image in (**d**) showed the details of a TiN particle.

Cross-sectional microstructures and energy- dispersive X-ray spectroscopy (EDS) mapping of the oxide layer after 1, 50, 100, and 200 hours of oxidation are shown in Fig. 4. There were 4 different regions identified in the cross-sectional microstructure, i.e. oxide layer (OL), internal oxidation depth (IOD), internal nitridation depth (IND) and substrate. According to EDS mapping, neither continuous Al_2_O_3_ nor Cr_2_O_3_ layer was found on the alloy surface after 200 hours of oxidation at 1000 °C. Instead, Al segregated near the top surface, while Cr distribution was almost uniform in the OL. The major component of the OL was CrTaO_4_ (Fig. 4(d) and Table 2, region A), and such a layer was apparently porous (Fig. 4(c,d), marked by red arrowheads). Furthermore, Al_2_O_3_ was dispersed within the IOD, and TiN were found within the IND (Fig. 4(d) and Table 2, region B). The size of TiN particles was around 1.5 μm, and they mainly formed in the dendrite regions.

**Table 2:** Chemical composition (in at %) of regions shown in Fig. 4(d).

ID#	N	O	Al	Si	Ti	Cr	Nb	Mo	Ta
A	—	69.0 ± 0.5	4.0 ± 0.8	0.2 ± 0.0	2.2 ± 0.3	10.8 ± 1.2	5.7 ± 0.6	1.1 ± 0.3	7.2 ± 0.1
B^*^	30.5 ± 3.6	6.9 ± 0.0	1.9 ± 0.0	1.1 ± 0.3	57.0 ± 2.7	0.7 ± 0.1	0.9 ± 0.3	0.7 ± 0.2	0.3 ± 0.1

^*^Determined with transmission electron microscope (TEM)-EDS.

Figure 2 shows the length of OL, IOD, and IND versus time with the corresponding oxidation weight gain at 1000 °C. Both OL and IND length increased significantly during the oxidation, while IOD length increment was moderate. This analysis indicates that exponential oxidation weight gain behaviour at 1000 °C was mainly attributed by a steady increase in the oxide layer formed externally and the nitridation depth.

### Oxidation behaviour at 1100 °C

**Figure 5:**
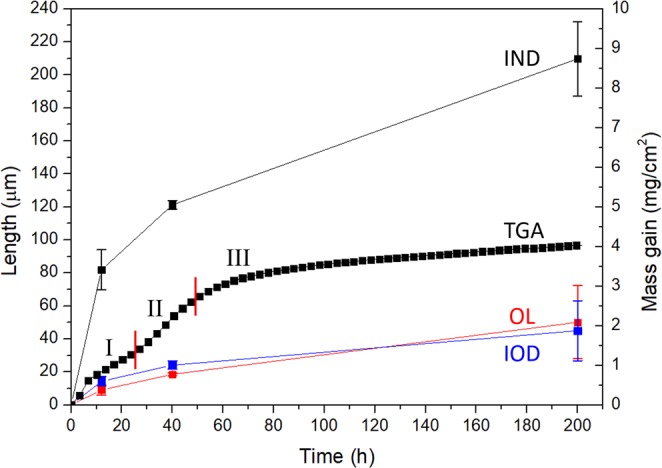
The oxidation mass gain (TGA curve) and length of OL, IOD, and IND at 1100 °C up to 200 hours. The error bars represented deviation from repeated measurements.

The oxidation mass gain curve of NV1 alloy at 1100 °C is shown in Fig. 5. It should be noted that, after 200 hours of oxidation, the mass gain per initial surface area was 4.03 mg/cm^2^, which was less than that of 1000 °C (6.53 mg/cm^2^). Three stages of mass gain dependence were identified from the oxidation mass gain curve, and they can be described by Eqs (2), (3) and (4), corresponding to Stage I, Stage II, and Stage III, respectively.

2 $${\rm{\Delta }}m=0.291{t}^{0.479}$$

3 $${\rm{\Delta }}m=0.058t$$

4 $${\rm{\Delta }}m=0.195{t}^{0.390}$$

**Figure 6:**
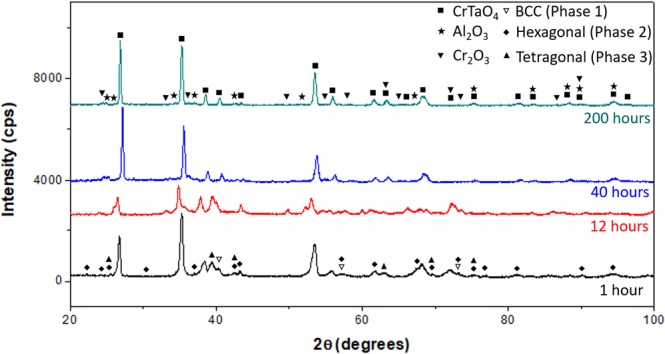
XRD patterns of NV1 alloy after oxidation at 1100 °C.

To understand the oxidation behaviour for different stages of mass gain dependence at 1100 °C for 200 hours, oxidised microstructures were characterized at the following intervals, i.e. 1, 12, 40, and 200 hours that respectively represented oxidation within the initial stage, Stage I, Stage II and Stage III. XRD patterns of the oxidised samples after each oxidation duration are shown in Fig. 6. The identified phases were identical to those obtained at 1000 °C (Fig. 3), and CrTaO_4_-based oxide was the major component of the oxide layer. The CrTaO_4_-based oxide detected after 200 hours of oxidation had lattice constants of *a* = 4.630 Å and *c* = 2.997 Å, which were 0.26% less in *a* axis and 0.76% less in *c* axis comparing to that of pure CrTaO_4_. The peaks from the substrate (BCC (Phase 1), Hexagonal (Phase 2), and Tetragonal (Phase 3) in Fig. 6) were visible after 1 hour of oxidation, then disappeared when the sample was oxidised for more than 12 hours.

**Figure 7:**
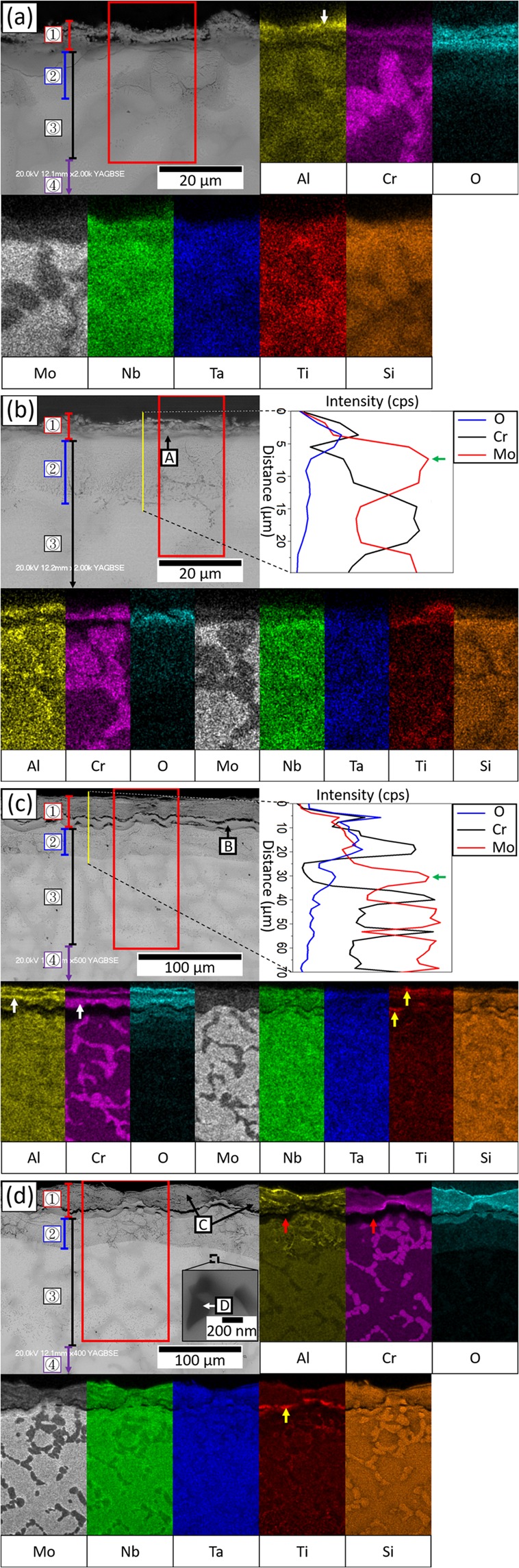
Cross-sectional BEI (grey scale images) and selected EDS mapping results (color-coded images) of oxide layers after (**a**) 1 (**b**) 12 (**c**) 40 (d) 200 hours of oxidation at 1100 °C. Note that 1, 2, 3, 4 in (**d**) respectively represent “OL”, “IOD”, “IND”, and “substrate”. For (**b**,**c**), EDS line-scan paths (yellow lines) and results (color-coded lines) were also provided. The enlarged images in (**d**) showed the details of a TiN particle.

After 1 hour of oxidation (Fig. 7(a)), the Al enrichment at some surface regions could be seen (Fig. 7(a), marked by a white arrowhead), while Cr and Ti distributed rather uniformly within the oxide layer. After 12 hours of oxidation (Fig. 7(b)), the external oxidation became more pronounced, and a 2 μm thick (Al, Cr)-depleted but Mo-rich zone was located at OL/IOD interface (Fig. 7(b) and Table 3, region A. The enrichment of Mo was marked by a green arrowhead in the line-scan result). After 40 hours of oxidation (Fig. 7(c)), (Al, Cr)-depleted but Mo-rich zone was significantly thickened (Fig. 7(c) and Table 3, region B. The Mo enrichment was marked by a green arrowhead in line-scan result), and laminar Al and Cr distribution in the OL became obvious (Fig. 7(c), marked by white arrowheads). In addition, relatively high Ti concentration was observed at the discrete surface areas and (Al, Cr)-depleted zone (Fig. 7(c), marked by yellow arrowheads). After 200 hours of oxidation (Fig. 7(d)), Ti enrichment at the (Al, Cr)-depleted zone became more obvious (Fig. 7(d), marked by a yellow arrowhead). The major constituent of the OL was CrTaO_4_-based oxide (Fig. 7(d) and Table 3, region C), which was similar to the results acquired at 1000 °C. Al_2_O_3_ was present in the IOD, and TiN particles (Fig. 7(d) and Table 3, region D) were found in the IND with a size of around 1.5 μm.

**Table 3:** Chemical composition (in at %) of regions shown in Fig. 7. The OL/IOD interface (Fig. 7(b), region A) was distinguished from other parts of oxide layer owing to the depletion of Cr and increase of Mo and Ti.

ID#	N	O	Al	Si	Ti	Cr	Nb	Mo	Ta
A	—	43.5 ± 7.9	4.3 ± 0.4	0.7 ± 0.1	10.0 ± 1.2	0.5 ± 0.1	9.7 ± 1.2	20.1 ± 1.5	11.2 ± 3.5
B	—	68.3 ± 0.4	1.6 ± 0.6	0.4 ± 0.1	5.9 ± 0.8	0.4 ± 0.1	7.1 ± 0.9	7.3 ± 0.4	9.0 ± 0.2
C	—	67.4 ± 0.9	6.5 ± 0.5	0.2 ± 0.0	3.2 ± 0.7	8.6 ± 0.2	5.9 ± 0.4	1.8 ± 1.0	6.4 ± 0.9
D^*^	45.0 ± 3.1	2.7 ± 0.8	0.1 ± 0.0	0.2 ± 0.3	50.9 ± 2.2	0.2 ± 0.0	0.5 ± 0.5	0.4 ± 0.4	0.0 ± 0.0

^*^Determined with TEM-EDS.

Figure 5 shows the length of OL, IOD, and IND versus time with respect to the oxidation weight gain at 1100 °C. Unlike the result of 1000 °C, only IND length increased drastically during the oxidation at 1100 °C, while OL and IOD length increments were moderate.

**Figure 8:**
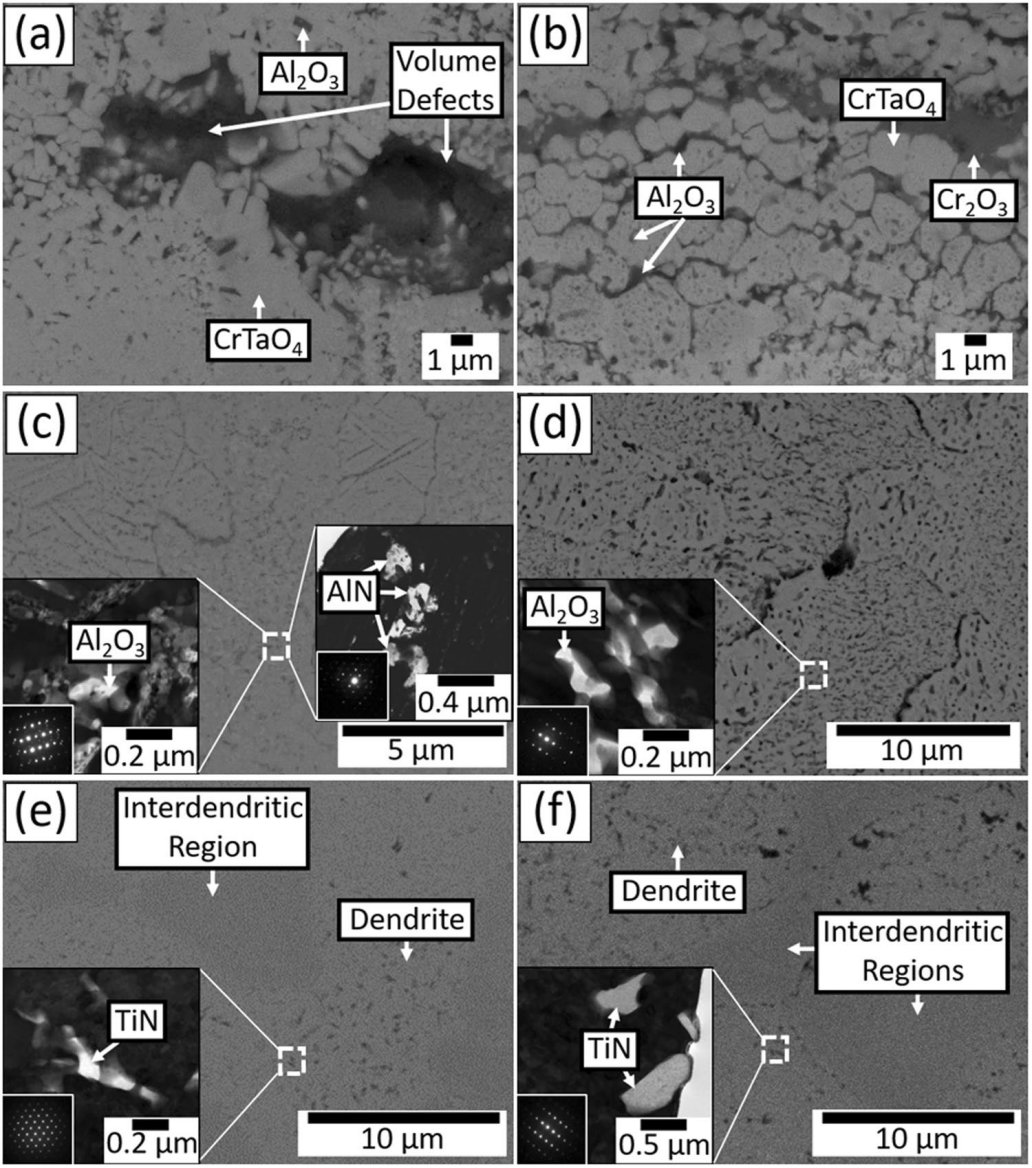
BEI and TEM bright field images of (**a**) external OL (**c**) IOD (**e**) IND at 1000 °C, (**b**) external OL (**d**) IOD (**f**) IND at 1100 °C. The beam incident directions for the selective area diffraction patterns (SADPs) are (**c**) [01-10] for Al_2_O_3_ and [01-11] for AlN (**d**) [01-12] (**e**) [011] (**f**) [−112].

In summary, the oxidation weight gain behaviour up to 200 hours was exponential at 1000 °C, whilst there were three stages at 1100 °C. Cross-sectional BEI and EDS mapping indicated large volume defects in the oxide scale (Fig. 4(c,d), and Fig. 8(a)), limited presence of (Al, Cr)-depletion zone, and thick OL and IND at 1000 °C. By contrast, a less porous oxide scale (Fig. 8(b)), and large IND were observed at 1100 °C. IOD observed in all oxidised samples contained Al_2_O_3_ as dominant oxides and minor AlN particles (Fig. 8(c,d)). In addition, TiN particles were found within IND in all oxidised samples, and they formed preferentially within the dendrites at both temperatures (Fig. 8(e,f)).

## Discussion

There were distinct differences in the weight gain behaviours of NV1 at 1000 and 1100 °C; XRD patterns and chemical compositions of all the oxidised samples indicated the major components of the oxide layers at both temperatures were CrTaO_4_-based oxide with dispersion of Al_2_O_3_ and Cr_2_O_3_. Since the distribution of Al_2_O_3_ and Cr_2_O_3_ were not continuous, neither Al_2_O_3_ nor Cr_2_O_3_ could provide effective barrier against oxidation in this study. However, it was previously reported that the CrTaO_4_ layer could improve the oxidation resistance of Ni-based superalloys by hindering the outward diffusion of elements from the substrate and reducing the oxygen inward diffusion rate to some extent^16–19^.

At 1000 °C, the oxidation kinetic curve of NV1 alloy followed exponential dependence. Such dependence is usually observed in either during the initial stage of oxidation or oxidation at relatively lower temperatures^20^. Since the exponential mass gain rate lasted for at least 200 hours, the later seems to be a more reasonable explanation. In other words, the reason for the exponential weight gain rate was due to the insufficient kinetics for NV1 to form protective oxide at 1000 °C, in this case CrTaO_4_-based oxide. Since the outward diffusion of elements was limited by insufficient kinetics, the inward diffusion of oxygen would become more dominant during the oxidation process. Little presence of Al and Cr depletion zones illustrated limited outward diffusion of both elements in NV1 alloy at this temperature, Fig. 4. Additionally, since the as-cast microstructure of NV1 alloy was composed of Mo-rich dendrite core and Cr- and Al-rich interdendritic region, the insufficient kinetics could result in heterogeneous oxidation among these chemically different regions. That is, Cr-rich and Al-rich region in interdendritic region could formed Cr_2_O_3_ and Al_2_O_3_, respectively, while Mo-rich dendrite core formed CrTaO_4_-based oxide. Such heterogeneous oxidation caused volume defects within the OL (Figs 4(c,d) and 8(a)) and prevented it from acting as an effective diffusion barrier. In Fig. 5, the OL length increment was significant and somewhat resembled the trend of overall weight gain. In addition, the IND length drastically increased with oxidation time. As a result, the weight gain of NV1 alloy at 1000 °C was mainly attributed to both oxidation and nitridation.

At 1100 °C, NV1 exhibited controlled oxidation with its parabolic weight gain behaviour, and the weight gain after 200 hours was smaller than that of 1000 °C. One may speculate that either evaporation of refractory oxide or severe spallation of OL may result in the loss of weight, however, there was neither significant loss of refractory contents nor noticeable spallation of the OL after the test. It appears that formation of denser CrTaO_4_ was responsible for hindering interdiffusion and provided certain degree of protection, Figs 7 and 8(b). Comparing the microstructure and EDS mapping results of samples acquired at 1000 °C and 1100 °C, the CrTaO_4_ layer formed at 1100 °C was denser than that formed at 1000 °C. Along with large (Al, Cr)-depleted zone observed at 1100 °C (Fig. 7(d), marked by red arrowheads), it is obvious that the kinetics of diffusion was enhanced, allowing more homogeneous growth of CrTaO_4_ at 1100 °C. In Stage I oxidation, homogeneous growth of CrTaO_4_ promoted the formation of a continuous CrTaO_4_ layer, which acted as a barrier for both inward diffusion of oxygen and outward diffusion of elements from the substrate. Since the elements from the substrate could not diffuse through CrTaO_4_ layer rapidly, they would accumulate beneath the CrTaO_4_ layer. These could explain why the external oxidation of Al at the surface barely developed further after 1 hour of oxidation at 1100 °C (Fig. 7(a)), as well as Mo and Ti segregation at the OL/IOD interface. In Stage II oxidation however, since CrTaO_4_-based mixed oxide layer was not an effective oxygen barrier, the oxidation process was not fully stopped. Al_2_O_3_ and Cr_2_O_3_ layers found within the oxide layer illustrated the progression of external oxidation front (Fig. 7(c), marked by white arrowheads). Further oxidation at OL/IOD interface also led to Mo evaporation since MoO_3_ would be highly volatile at above 800 °C^21^. The evaporation of MoO_3_ created additional paths for oxygen ingress, accelerating the oxidation of the regions beneath the oxide layer. But since MoO_3_ evaporated only from ~2 μm-thick OL/IOD interface, the weight loss resulting from MoO_3_ evaporation shall be neglectable comparing to the weight gain from the accelerated oxidation of the other regions. Consequently, the net oxidation weight gain rate was increased. But eventually, the oxidation process would slow down due to additional formation of CrTaO_4_ layer in Stage III oxidation.

The CrTaO_4_-based oxide layer observed in present study showed different lattice constants and compositions at 1000 and 1100 °C. It mainly contained 2.5 at% more Al and 2.2 at% less Cr when the testing temperature increased from 1000 °C to 1100 °C, which also resulted in the change of lattice constants. The increased Al content in CrTaO_4_-based oxide at 1100 °C reflected the fact that the kinetics for Al was enhanced when the temperature was increased from 1000 °C to 1100 °C, allowing more prominent outward diffusion of Al into the CrTaO_4_-based oxide layer. The reduced Cr content was most likely caused by substitution of Cr atoms with Al atoms in CrTaO_4_ lattice since both Al and Cr could form rutile-type AlTaO_4_ and CrTaO_4_. Furthermore, the observed CrTaO_4_-based oxide contained up to 7 at% of Al, 0.2 at% of Si, 3.9 at% of Ti, 6.3 at% of Nb, and 2.8 at% of Mo, along with roughly 10 at% reduction in O and negative deviations in lattice constants comparing to the pure CrTaO_4_. All the results described above illustrated the non-stoichiometric nature of CrTaO_4_. Such property potentially suppressed the formation of detrimental oxides, such as Nb_2_O_5_ and TiO_2_, since the metallic atoms of these oxides were accommodated by CrTaO_4_ instead of forming their individual oxides. However, since CrTaO_4_-based oxide also contained up to 7 at% of Al, it might also hinder the rapid formation of a protective Al_2_O_3_ layer by solutioning outward-diffusing Al atoms and preventing their oxidation. Nevertheless, the CrTaO_4_-based oxide layer protected NV1 alloy from catastrophic oxidation by hindering the outward diffusion and selective oxidation of refractory elements, as well as reducing the ingress of oxygen to some extent, which consequently led to the formation of IOD with Al_2_O_3_ dispersion (Fig. 8(c,d)) since the oxygen partial pressure in the IOD was too low for the formation of other oxides.

The existence of TiN (cubic, *a* = 4.230 Å) and minor AlN (hexagonal, *a* = 3.119 Å and *c* = 5.026 Å) within the IND was confirmed by both TEM-SADPs (Fig. 8) and literature^22^. The greater length of IND over IOD indicated the ingestion of nitrogen was faster than oxygen, allowing the partial pressure of nitrogen to reach the critical value for TiN formation in a wider region than that of the partial pressure of oxygen to reach the critical value for Al_2_O_3_ formation. Furthermore, TiN particles were found to present at dendritic regions at both temperatures, Fig. 8(e,f). In a previous study on the oxidation and nitridation behaviour of Cr-Si system^23^, it has been suggested that Si addition generally enhances nitridation resistance of pure Cr. In present study, since no obvious trace of TiN was found within Si-bearing Cr- rich phase, it is reasonable to assume that nitrogen preferentially diffused through Mo-rich dendrites and formed TiN particles, while interdendritic regions were less affected by nitrogen. In previous study on the oxidation of Ni- base superalloys, TiN could be found beneath the internal oxidation layer^24–26^, and the solubility and diffusion of nitrogen was reported to increase with increasing Cr content in Ni-Cr-Al-Ti superalloy without Si addition^25^. Therefore, both Cr and Si content might be important for providing nitridation resistance. Although the exact nitrogen content was not determined in this work, it is still clear that nitridation in RHEAs can occurred during high temperature exposure, and its influences on the properties of RHEAs could be an important subject for further investigation.

**Figure 9:**
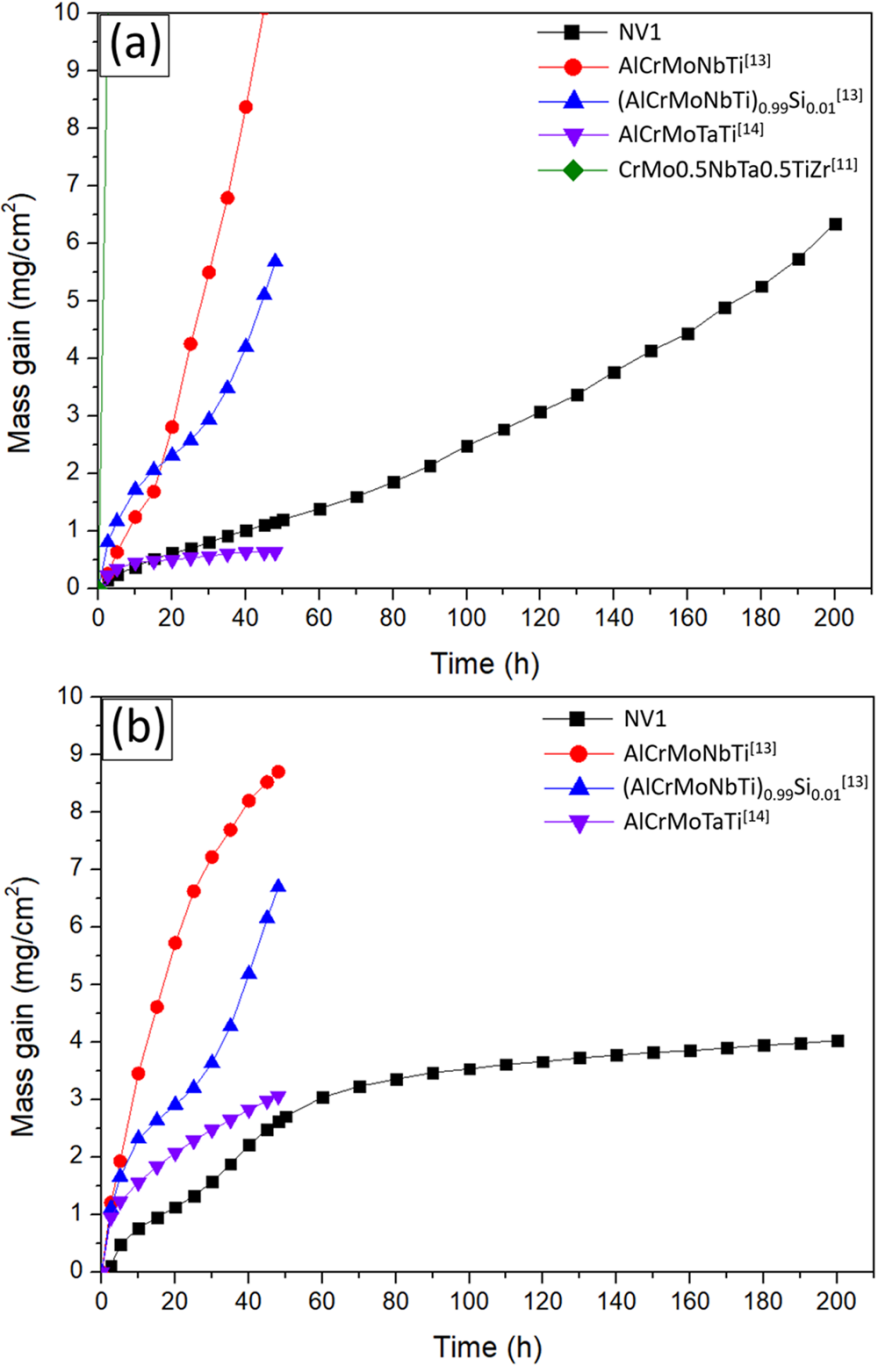
Comparison of oxidation weight gain between NV1 alloy and selected RHEA/RCCAs at (**a**) 1000 (**b**) 1100 °C.

Comparisons between NV1 with several advanced RHEA/RCCAs are shown in Fig. 9. Comparing to the oxidation behaviour of CrMo_0.5_NbTa_0.5_TiZr alloy at 1000 °C reported by Senkov *et al*.^11^, CrMo_0.5_NbTa_0.5_TiZr suffered from extremely high mass gain because no protective oxide layer was formed on the alloy surface during the oxidation. A comparison between the oxide layer of CrMo_0.5_NbTa_0.5_TiZr alloy and present work reveals that NV1 alloy had tendency of selective oxidation to form Al_2_O_3_, Cr_2_O_3_ and CrTaO_4_-based oxide, which led to better oxidation resistance. Furthermore, NV1 benefited from Si addition to resist nitridation to certain extent at the interdendritic regions. So, the weight gain of NV1 during high temperature exposure was significantly less. As for more recently developed oxidation-resistant RHEAs with similar constituents comparing to NV1 alloy, the performance of NV1 alloy was superior to AlCrMoNbTi^13^ and fell between (AlCrMoNbTi)_0.99_Si_0.01_^13^ and AlCrMoTaTi^14^ at 1000 °C within 48 hours. At 1100 ^o^C, NV1 alloy outperformed all 3 other alloys (AlCrMoNbTi, (AlCrMoNbTi)_0.99_Si_0.01_, and AlCrMoTaTi) within 48 hours. The main difference in the composition of NV1 alloy was 75% less Ti comparing to AlCrMoNbTi, (AlCrMoNbTi)_0.99_Si_0.01_, and AlCrMoTaTi. Since CrTaO_4_ layer played a major role in reducing the oxidation of NV1 alloy, it is very likely that the reduced Ti content of NV1 alloy promoted the formation of CrTaO_4_ layer, leading to enhanced oxidation resistance at 1100 °C. These previous works suggested that parabolic mass gain rate observed for the oxidation of RHEAs/RCCAs could be attributed to the formation of protective Al_2_O_3_ and Cr_2_O_3_ on the alloy surface, although no detailed microstructural characterization were shown in these studies^13,14^. However, the testing duration in these previous studies was within 48 hours, which was not long enough to confirm whether these RHEAs/RCCAs were really protected by Al_2_O_3_ and Cr_2_O_3_. To study protective oxide scale, testing duration of 100 hours or beyond is necessary^27–29^. Calculations of Al activities with TCHEA 3.0 database in Thermo-Calc software (version 2018b) revealed the Al activity of NV1 alloy was 1 × 10^−5^ at 1000 °C and 2 × 10^−5^ at 1100 °C, while AlCrMoTaTi alloy^14^ had Al activity of 3.63 × 10^−6^ at 1000 °C and 4.99 × 10^−6^ at 1100 °C. Obviously, NV1 alloy possessed much higher Al activities at both temperature than those of AlCrMoTaTi alloy, which was claimed to be able to form a protective Al_2_O_3_ layer, but no protective Al_2_O_3_ layer was found on NV1 alloy in present study. This work clearly demonstrates that the formation of CrTaO_4_-based oxide dominated the oxidation behaviour and attributed to parabolic mass gain behaviours at 1100 °C. However, protection from CrTaO_4_-based oxide could not fully prevent the ingestion of oxygen and nitrogen, and the increase of testing duration enabled changes in weight gain behaviours due to further internal oxidation and nitridation. Previous study on RHEAs/RCCAs^13^ even suggested cracked oxide at the corners of the sample could affect the weight gain behaviours^30,31^, which was not observed in present study.

In summary, isothermal oxidation at 1000 and 1100 °C was performed on a novel RHEA for up to 200 hours, such long-term oxidation test has not been reported before for this class of alloys. The weight gain behaviours during the tests could be affected by evolutions of external oxidation, internal oxidation, and nitridation. The multiple stages of weight gain behaviour at 1100 °C were never discussed in previous studies since most of them only presented the results of oxidation up to 48 hours^12–14,32^. We believe that CrTaO_4_-based protection layer and Si addition may be important for future development of RHEA against oxidation and nitridation. However, it is also possible that the growth of a protective Al_2_O_3_ or Cr_2_O_3_ layer was hindered by prominent CrTaO_4_-based oxide presence. In both cases, the nature of such CrTaO_4_-based oxide needs to be carefully examined in the future studies of RHEAs.

This work has provided an insight on the formation of CrTaO_4_-based external oxide on a novel RHEA, and how it could influence the weight gain behaviours for up to 200 hours. The pre-oxidation sample contained dendritic microstructure, with Mo-rich phase as dendrite core and Cr-rich phase, minor Al-rich phase located in interdendritic region. Oxidation mechanisms have been deduced based on experimental observations. The external oxides were mainly CrTaO_4_-based with dispersion of Al_2_O_3_ and Cr_2_O_3_. The external oxides at 1000 °C contained large volume defects due to insufficient kinetics to form dense CrTaO_4_-based oxide layer, resulting in an exponential dependence of weight gain throughout 200 hours of testing duration. At 1100 °C, mass gain curve showed three different stages of dependence during 200 hours of oxidation. TiN particles were mainly found in dendritic regions. The weight gain of the alloy at 1100 °C after 200 hours was 4.03 mg/cm^2^, which indicates that it is one of the most oxidation resistant RHEAs according to literature data to-date.

## Methods

The alloy of interest contains Al, Cr, Mo, Nb, Si, Ta, Ti; it is designated as NV1, and its composition is given in Table 1. The design of NV1 was based on CrMo_0.5_NbTa_0.5_TiZr alloy proposed by Senkov *et al*.^11,33^; the addition of Al, Si and further increase in Cr content were intended to promote the formation of protective oxide layer on the alloy surface, while Ti content was reduced and Zr was removed to minimize possible competitive oxide formations of TiO_2_ and ZrO_2_^34^. The ingot of NV1 was prepared by the cold crucible levitation melting method with high purity elemental raw materials (>99.9 wt%), and a 1.1 kg cone-shaped ingot was cast. Chemical compositions were determined with Shimadzu Lab Center XRF-1800 and JEOL JXA-8900R. The compositions listed in this work were measured with EPMA unless otherwise stated. The ingot was machined by electrical discharge machining for experimental analysis.

X-ray diffraction (XRD) analysis was conducted to identify phases. Sample dimension was 10 × 10 × 2 mm^3^, with surfaces ground to 1200 grit with SiC abrasive paper. The as-cast and oxidised samples were analysed by a Rigaku MiniFlex 600X-ray diffractometer. Radiation source was Cu Kα, and the goniometer scanning was performed between 2θ = 20–100° with a scanning rate of 10° per minute. Oxidation mass gain curves were measured at 1000 °C and 1100 °C with a thermogravimetric analyser (TGA) - SETARAM TAG 24-18S simultaneous symmetrical thermoanalyser; specimens for TGA had a dimension of 6 × 3 × 2 mm^3^ with each surface ground to 1200 grit with SiC abrasive paper, and tests were performed with flowing dry air; the sample was hung by Pt wire in a vertical furnace, and the weight change was recorded automatically every 0.04 hour up to 200 hours at 1000 °C and 1100 °C. The measured curves were expressed with fitted equations with R^2^ values greater than 0.99. In order to study oxidation mechanisms, additional isothermal oxidation tests were performed with muffle furnace in the atmosphere. Dimension and surface roughness of samples for isothermal oxidation tests were the same as those for XRD analysis, and the tests were conducted for 1, 50, 100, and 200 hours at 1000 °C, and 1, 12, 40, 200 hours at 1100 °C. The choice of testing durations of isothermal oxidation tests was associated with the oxidation behaviours determined with TGA. Oxidised samples were removed from the furnace once the testing duration was reached, and subsequently air-cooled to room temperature. The isothermal oxidation test was repeated at least 4 times for each testing condition, and the microstructure of the post- test specimens was compared to that of the TGA-tested specimens to confirm the consistency of the experimental results. Microstructures and composition of oxidised samples were analysed by Shimadzu EPMA-1610, JEOL JXA-8500F, JEOL JSM-7610F, and Hitachi S-4700 scanning electron microscope equipped with EDS and backscatter electron detector. Samples for TEM were prepared by means of focused ion beam method with SEIKO SMI 2050 and observed with JEOL JEM-F200.
